# Unlocking Glioblastoma Vulnerabilities with CRISPR-Based Genetic Screening

**DOI:** 10.3390/ijms25115702

**Published:** 2024-05-23

**Authors:** Yitong Fang, Xing Li, Ruilin Tian

**Affiliations:** 1 Department of Medical Neuroscience, School of Medicine, Southern University of Science and Technology, Shenzhen 518055, China; 12010236@mail.sustech.edu.cn (Y.F.); 12031244@mail.sustech.edu.cn (X.L.); 2 Key University Laboratory of Metabolism and Health of Guangdong, Southern University of Science and Technology, Shenzhen 518055, China

**Keywords:** glioblastoma, CRISPR-based genetic screening, therapy resistance, genetic determinants, therapeutic targets

## Abstract

Glioblastoma (GBM) is the most common malignant brain tumor in adults. Despite advancements in treatment, the prognosis for patients with GBM remains poor due to its aggressive nature and resistance to therapy. CRISPR-based genetic screening has emerged as a powerful tool for identifying genes crucial for tumor progression and treatment resistance, offering promising targets for tumor therapy. In this review, we provide an overview of the recent advancements in CRISPR-based genetic screening approaches and their applications in GBM. We highlight how these approaches have been used to uncover the genetic determinants of GBM progression and responsiveness to various therapies. Furthermore, we discuss the ongoing challenges and future directions of CRISPR-based screening methods in advancing GBM research.

## 1. Introduction

Glioblastoma (GBM) is the most common malignant brain tumor in adults [1,2,3], with an annual incidence rate of approximately 3.19/100,000 [4]. GBM is highly aggressive, with a median survival time of less than 2 years and a dismal 5-year survival rate of less than 6% even following standard multimodal therapy [5,6]. The current standard of care involves maximally safe surgical resection, followed by radiotherapy and chemotherapy [7]. However, GBM commonly develops robust resistance to these treatments due to its high degree of intrinsic plasticity and genomic/phenotypic heterogeneity, posing significant challenges for effective therapy and contributing to poor prognosis [1,8,9]. Understanding the molecular mechanisms underlying treatment resistance in GBM is urgently needed to enable the development of more effective, targeted strategies to improve patient outcomes.

High-throughput functional genomics screening, including RNAi- and CRISPR-based screening, is a powerful approach to elucidate disease mechanisms and identify new therapeutic targets [10,11,12,13]. In this review, we discuss the latest insights from CRISPR screening studies of GBM, focusing on discoveries related to GBM progression and responsiveness to radiotherapy, chemotherapy, and immunotherapy. We also outline the challenges and opportunities for further development of CRISPR-based screening approaches to advance GBM research.

## 2. Therapy Resistance in GBM

The current standard of care (SOC) for GBM involves a multimodal approach consisting of surgical resection, radiation therapy, and chemotherapy [7,14,15]. Surgery aims to remove as much of the tumor as possible, but complete resection is often challenging due to the invasive nature of GBM cells [7,15]. Following surgery, radiation therapy is administered to target any remaining tumor cells in the surrounding brain tissue [7]. Concurrently, chemotherapy, typically with temozolomide (TMZ), is administered to enhance the overall treatment efficacy [14,15]. The median overall survival with the current SoC is approximately 14–16 months, compared to 12 months with radiotherapy alone [16]. However, despite aggressive treatment, GBM remains highly resistant to therapy, leading to disease recurrence [15]. Several mechanisms have been implicated in the therapy resistance of GBM. First, GBM cells display inherent phenotypic plasticity, allowing them to dynamically adapt to different microenvironments and therapeutic pressures [1]. This plasticity is driven by genetic mutations, epigenetic modifications, and interactions with the tumor microenvironment [1]. For example, GBM cells can acquire resistance through the activation of DNA repair pathways, such as the DNA damage response (DDR) pathway [17]. GBM cells can upregulate DNA repair proteins, such as the methyl guanine methyl transferase (MGMT), enabling them to efficiently repair therapy-induced DNA damage, thereby reducing the effectiveness of radiation and chemotherapy [18,19,20,21]. Furthermore, the overexpression of drug efflux pumps, such as ATP-binding cassette (ABC) transporters, allows GBM cells to actively pump out chemotherapeutic agents, limiting their intracellular accumulation and effectiveness [22]. The tumor microenvironment also plays a significant role in therapy resistance, as it can create a protective niche for GBM cells, promote angiogenesis, and suppress immune responses [9,18].

GBM also exhibits marked intra-tumoral heterogeneity, with the presence of distinct subpopulations of tumor cells harboring diverse molecular profiles and therapeutic sensitivities [1]. This heterogeneity poses challenges for effectively targeting all tumor cell populations. Glioblastoma stem cells (GSCs) are a subpopulation of GBM cells with stem cell-like characteristics of self-renewal and differentiation [23,24]. They exhibit intrinsic resistance to various treatment modalities, such as chemotherapy and radiation therapy, making them a key factor in treatment failure and disease recurrence [1,17,25].

The complex interplay of these resistance mechanisms underscores the formidable challenge of effectively treating GBM and highlights the need for innovative therapeutic approaches targeting therapy-resistant GBM cells.

## 3. CRISPR/Cas9-Based Genome Engineering Tools

**Figure 1:**
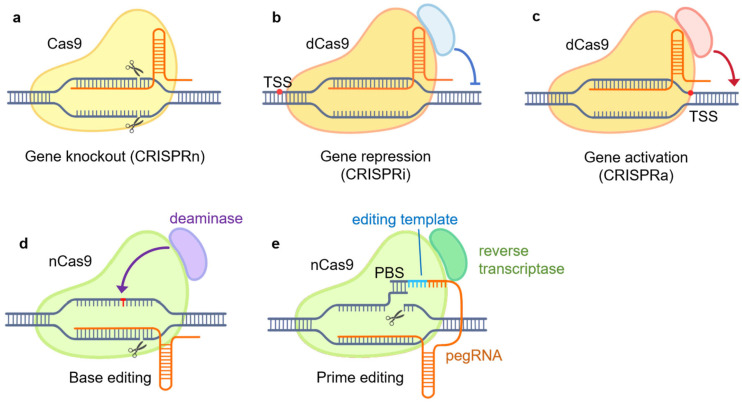
CRISPR/Cas9-based genome engineering tools. (**a**) CRISPR knockout (CRISPRn): gene knockout by targeting Cas9 to specific sites, inducing DNA double-stranded breaks (DSBs) and non-homologous end joining (NHEJ) repair. DSB is caused by the native DNA-cleavage activity of Cas9; (**b**) CRISPR interference (CIRSPRi): gene repression by targeting the dCas9–repressor complex to specific gene promoters; (**c**) CRISPR activation (CRISPRa): gene induction by targeting the dCas9–activator complex to promoter regions; (**d**) base editing: the nCas9–deaminase complex is utilized to convert a DNA base to another directly without DSB; (**e**) prime editing: the nCas9–reverse-transcriptase complex and pegRNA is utilized to introduce targeted genetic modifications without DSB. PBS, primer-binding site; dCas9, dead Cas9, inactive Cas9; nCas9, Cas9 nickase; pegRNA, prime editing guide RNA.

Regularly Interspaced Short Palindromic Repeats (CRISPR) and CRISPR-associated protein 9 (Cas9) systems have revolutionized the field of genetics and molecular biology [26,27,28,29] (Figure 1). Originally discovered as part of a bacterial adaptive immune system, the CRISPR-Cas9 system offers unprecedented precision and versatility in genome editing and engineering [30,31]. With the rapid progress in CRISPR technologies, a wide array of genome manipulation tools have emerged. In this section, we will provide an overview of several commonly employed CRISPR-based tools.

CRISPR knockout, or CRISPRn, utilizes the native DNA-cleavage activity of Cas9 guided by single-guide RNA (sgRNA) to introduce double-stranded breaks (DSBs) at specific genomic loci [32]. Subsequently, the cell’s DNA repair mechanisms, such as non-homologous end joining (NHEJ), attempt to repair the breaks [28,32,33]. However, the repair often results in insertions or deletions that can cause frameshift alterations and knockout of the target gene product through premature stop codons or altered reading frames [32,34]. CRISPRn provides a straightforward tool to investigate gene function through loss-of-function mutations analogous to traditional knockout approaches.

CRISPR interference, or CRISPRi, employs a catalytically inactive Cas9 (dead Cas9, dCas9) protein fused to transcriptional repressors or epigenetic modifiers [35]. By guiding the dCas9–repressor complex to specific gene promoters with sgRNAs, CRISPRi allows for precise gene silencing in a temporally controlled, reversible manner compared to permanent knockout [26,28,36].

CRISPR activation, or CRISPRa, uses dCas9 fused with transcriptional activation domains [28]. By targeting the dCas9–activator complex in promoter regions, CRISPRa enables the upregulation of endogenous gene expression, providing a means to probe gene function through overexpression complementary to CRISPRi [28,37].

Base editing enables the direct conversion of one DNA base to another without inducing double-strand breaks (DSBs) [32]. It relies on the fusion of a Cas9 nickase (nCas9) protein with a base-modifying enzyme, such as cytidine deaminase or adenine deaminase. By guiding the nCas9 complex to specific genomic loci using sgRNAs, base editing allows the introduction of specific point mutations or the correction of disease-associated genetic variants [32,34]. This approach provides a versatile tool for investigating the functional consequences of specific genetic changes.

Prime editing represents a recent advancement in CRISPR technology that allows more versatile genome editing without DSBs [32]. It utilizes a nCas9 fused with an engineered reverse transcriptase, along with a prime editing guide RNA (pegRNA) that both specifies the target site and provides the template for new DNA synthesis. Prime editing allows for the precise insertion, deletion, or substitution of DNA sequences at specific genomic sites [32,38].

## 4. CRISPR/Cas9-Based Functional Genomics Screening Strategies in GBM

Functional genomics screening is a powerful approach for systematically elucidating gene function and identifying genetic determinants of cellular processes on a genome-wide scale. Traditional functional screens have relied on RNA interference (RNAi), using short hairpin RNA (shRNA) or small interfering RNA (siRNA) libraries [10]. The development of CRISPR/Cas9 technologies has revolutionized functional screening by significantly improving the robustness and scalability of the screens [10,27].

**Figure 2:**
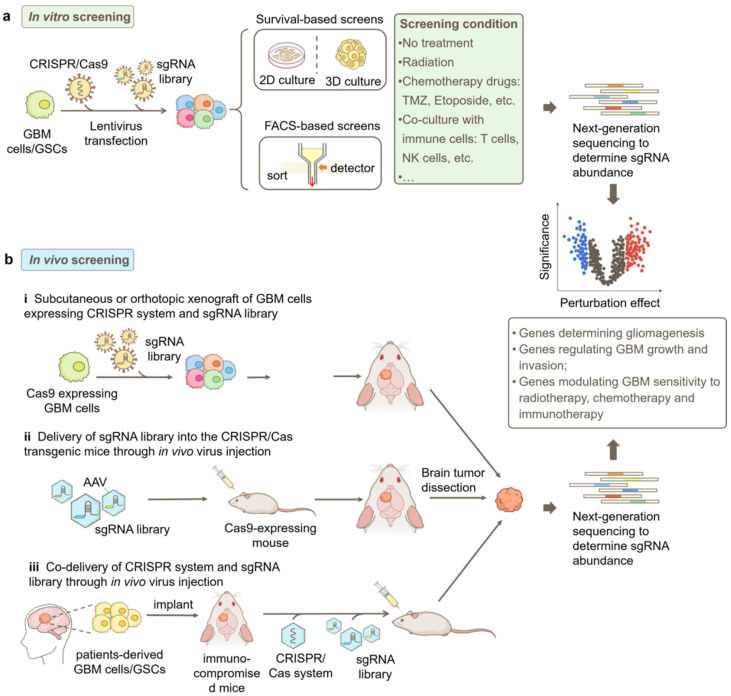
Strategies for CRISPR-based genetic screens in GBM research. (**a**) Workflow of a typical in vitro screen. GBM cells or patient-derived GSCs are engineered to stably express the CRISPR/Cas9 system, and the sgRNA library is transduced into the cells by lentivirus. Subsequently, the cells can be cultured in 2D or 3D and selected based on the phenotype of interest, such as cell responsiveness to selection pressures such as radiation or chemotherapy treatment and fluorescent signals from genetically encoded reporters, chemical probes, or immunofluorescence staining. (**b**) Different approaches for in vivo screens in GBM, including i. subcutaneous or orthotopic implantation of GBM cells that express the CRISPR/Cas9 system and sgRNA library into mice; ii. AAV-delivery of sgRNA library into the brain of CRISPR/Cas transgenic mice; and iii. co-delivery of the CRISPR/Cas9 system and sgRNA library into patient-derived xenograft (PDX) GBM models. Next-generation sequencing (NGS) is used to quantify sgRNA abundances in collected samples for both in vitro and in vivo screens. Bioinformatic analysis identifies genes modulating GBM phenotypes based on sgRNA enrichment or depletion. FACS, fluorescence-activated cell sorting.

Various CRISPR screening strategies have been employed to date in GBM research. Most commonly, the screens are conducted in vitro using GBM cell lines or patient-derived glioblastoma stem cells (GSCs). Typically, the cells are genetically engineered to stably express a specific CRISPR system, usually CRISPRn, CRISPRi, or CRISPRa [33]. To introduce genetic perturbations, a genome-wide sgRNA library or a focused sgRNA library targeting a specific set of genes is delivered into the cells via lentiviral transduction [39] (Figure 2). Subsequently, the transduced cells are selected according to the phenotype of interest, either by their fitness under selection pressures such as radiation or chemotherapy treatment or by fluorescence-activated cell sorting (FACS) based on fluorescent signals from genetically encoded reporters, chemical probes, or immunofluorescence staining [33]. After selection, genomic DNA is extracted from cells in different groups. Next-generation sequencing (NGS) and bioinformatics analysis are performed to obtain the abundances of sgRNAs and determine the effect and significance of each genetic perturbation on the phenotype of interest (Figure 2).

In addition to in vitro screens, in vivo screens have emerged as valuable approaches to studying GBM in a more physiologically and pathologically relevant context [40]. Compared to in vitro screens, in vivo screens offer significant advantages in providing the intricate cellular and molecular milieu of GBM tumors, facilitating the understanding of complex interactions between genes and the tumor microenvironment [41].

In vivo screens in GBM can be conducted through multiple approaches (Figure 2b). One common method involves the subcutaneous or orthotopic implantation of GBM cells that express the CRISPR/Cas9 system as well as the sgRNA library into mice [37,40]. The sgRNA abundance in cells prior to implantation and in tumors harvested at different time points after implantation can be determined by NGS to identify genes involved in GBM progression in vivo. Additionally, these implanted mice can be treated with clinically relevant therapies to screen for genes responsible for treatment responsiveness [41].

Another approach is to utilize genetically engineered mice that express Cas9 in GBM-relevant cell types, such as astrocytes [37,41,42]. In this method, the sgRNA library can be delivered via adeno-associated virus (AAV) directly into the brain of healthy mice to study tumorigenesis or into the brain of mice bearing primary tumors to study tumor progression [29,41].

Furthermore, patient-derived xenograft (PDX) models [29] can be potentially employed for in vivo screens in GBM. A promising approach involves the implantation of GBM patient tumor samples into immunocompromised mice. Subsequently, the CRISPR-Cas9 system and sgRNA library are delivered via AAV injection. This method would enable the examination of gene perturbations in a more clinically relevant context and facilitate the development of personalized therapies.

## 5. CRISPR/Cas9-Based Genetic Screening in GBM

### 5.1. GBM Progression

CRISPR screens have been utilized to investigate multiple steps of GBM progression, including tumorigenesis, tumor growth, and tumor invasion. In an effort to uncover genetic factors that regulate GBM tumorigenesis, Chow et al. developed an AAV-mediated direct in vivo CRISPR screen approach [42]. They injected an AAV library targeting tumor suppressor genes commonly mutated in human cancers into the brains of mice expressing Cas9 conditionally in astrocytes. Through this approach, they identified distinct mutational profiles across tumors and co-occurring driver combinations like *B2m-Nf1* and *Zc3h13-Rb1* in GBM.

GSCs are thought to be the root of GBM growth. Toledo et al. performed pioneering genome-wide CRISPRn screens in patient-derived GSCs and human neural stem cells (NSCs), looking for genes that are essential for growth and survival, specifically in the GSCs but not the NSCs [43]. Several hit genes were identified, both those specific to individual patient samples and those shared across samples. Follow-up experiments validated *PKMYT1* as a shared hit that is redundantly required with *WEE1* for proper cell division in NSCs but whose redundancy is lost in GSCs, making them uniquely vulnerable to *PKMYT1* inhibition. This suggests *PKMYT1* could be a promising therapeutic target for GBM. A subsequent comprehensive study by MacLeod et al. performed genome-wide CRISPRn screens in a panel of 10 patient-derived GSCs to identify core fitness genes across GSCs as well as genotype-specific vulnerabilities [44]. They identified transcription factors *SOX2* and *SOX9*, histone methyltransferase *DOT1L*, and cytokine signaling suppressor *SOCS3* as important regulators of stemness and GSC fitness. Stress response pathways like the ufmylation pathway and the ER-associated degradation (ERAD) pathway were also revealed to be important. Validation experiments confirmed the role of transcription factor JUN and its upstream kinase MAP2K7 in promoting GSC growth [44].

The tumor microenvironment plays a crucial role in supporting the growth and maintenance of GSCs. Tang et al. developed a 3D bioprinted tissue model containing GSCs and stromal cells to study their interactions in a physiologically relevant microenvironment [45]. The authors performed parallel whole-genome CRISPR-Cas9 loss-of-function screens in GSCs cultured as spheres as well as in the 3D bioprinted tissue model to identify context-specific functional dependencies. They found both common essential genes, like those involved in translation and DNA repair, as well as context-specific genes. Genes related to cell cycle, metabolism, and hypoxia response were more essential in spheres, while genes involved in transcription, development, and NF-κB signaling depended more on the 3D microenvironment. Two novel hit genes identified from the 3D screen, *PAG1* and *ZNF830*, were validated to be essential in both the 3D model and mouse xenografts when knocked out via CRISPR, suggesting they may be potential therapeutic targets for glioblastoma [45].

In addition to genome-wide screens, targeted screens have been conducted in GBM to investigate specific domains of biology related to GBM growth. Epigenetic alterations are pervasive in GBM, contributing to GBM progression. To identify epigenetic regulators controlling GBM growth, Ozyerli Goknar et al. conducted CRISPRn screens in GBM cell lines U373 and T98G, using an epigenetic-focused sgRNA library that targets chromatin modifiers [46]. They identified several novel essential genes, including *ASH2L*, *RBX1*, and *SSRP1*. Further analysis revealed that ASH2L interacts with histone methyltransferases SETD1A, SETD1B, MLL1, and MLL2, and its depletion led to the downregulation of cell cycle genes. Depletion of *ASH2L* also inhibited tumor growth in mouse models. In another study, Qiu et al. performed gene expression analysis and genome-wide CRISPRn screens in a panel of patient-derived GSCs and normal NSC [24]. They identified chromatin regulators that are selectively dependent on GSCs, including the transcription factor YY1. Further analysis revealed that YY1 regulates RNA polymerase II transcription and RNA N6-Methyladenosine(m^6^A) programs in GSCs by controlling chromatin loops and interacting with the transcriptional CDK CDK9. Knockdown or inhibition of *YY1* or CDK9 triggered an interferon response program through RNA m^6^A modification and synergized with immunotherapy in mouse glioma models. Another focused screen targeting 557 E3 ligases in GBM cell line U87 identified RNF185 as a tumor suppressor by inhibiting proliferation, migration, and inducing apoptosis [47]. Mechanistically, the study found that *RNF185* expression is reduced in glioma due to promoter hypermethylation and increased expression of the oncogenic miRNA miR-587, which directly targets the 3‘UTR of *RNF185*.

In addition to coding genes, long non-coding RNAs (lncRNAs) have also been found to play important roles in GBM progression [48,49,50]. To systematically probe lncRNA functions in GBM, Zheng et al. conducted CRISPRi screens in GBM cell lines U251 and U87, using a sgRNA library targeting lncRNAs that are dysregulated in GBM [49]. They identified that the lncRNA *DARS1-AS1*, which is highly expressed in GBM tumors, is essential for GBM growth. Mechanistically, *DARS1-AS1* was found to interact with the RNA-binding protein YBX1 and promote its function in stabilizing mRNAs of key regulators of cell cycle progression, self-renewal, and homologous recombination (HR)-mediated DSB repair. An in vivo screen focusing on lncRNA function in GBM has also been reported [48]. This screen identified 17 lncRNA hits that are distinct from in vitro screens.

Tumor invasion is another key driver of GBM progression. A transwell assay has been used in a focused CRISPRn screen to identify regulators of GBM invasion [51]. The screen uncovered *MAP4K4*, which was validated to be important for migration in additional assays and cell lines. Further experiments showed that *MAP4K4* inhibition or knockout reduced migration in vitro and in human tumor slices. *MAP4K4* expression correlated with epithelial-mesenchymal transition markers, and its loss drove cells toward a non-invasive state. Garcia et al. developed more sophisticated invasion models using 3D hydrogel [52]. A CRISPR screen targeting metabolic genes in the hydrogel models identified cystathionine gamma-lyase (CTH) as essential for GBM invasion. CTH is the rate-limiting enzyme in the transsulfuration pathway that generates the antioxidant cysteine [52,53]. Knockdown or inhibition of CTH impaired GBM invasion in vitro and in vivo and caused cysteine deficiency and ROS accumulation, effects that were rescued by cysteine supplementation [52].

CRISPR screening has also been employed to investigate other aspects of GBM progression. For example, Tu et al. used CRISPR screening to identify genetic vulnerabilities in GBM-carrying telomerase reverse transcriptase (TERT) promoter mutations (TPMs), a genomic alteration present in over 80% of GBM cases [54]. They showed that while TPM status correlated with differential gene expression and dependencies on ETS transcription factors like *ELF1*, *ETV4*, and *GABP*, it did not specifically correlate with TERT dependency.

### 5.2. Responsiveness to Radiotherapy

While radiotherapy kills GBM by causing irreparable DNA damage, tumor cells can acquire resistance through enhanced DNA repair, dysregulated cell cycle checkpoints, evasion of apoptosis, and activation of pro-survival signaling pathways that counteract radiation-induced cell death [55].

To identify genes that mediate radioresistance in GBM, Zhu et al. conducted a genome-wide CRISPRa screen and identified *CARHSP1*, *KIAA0895*, *FBMIL1*, and *STRA6* as top hits that promoted radioresistance when overexpressed [56]. Mechanistically, they found *CARHSP1* levels were upregulated in irradiation-resistant cells, and its overexpression activated the TNF-α inflammatory pathway to mediate radioresistance.

In another study, Liu et al. focused on the role of lncRNA in GBM radioresistance. Through a CRISPRi screen targeting over 5000 lncRNA loci in GBM cells, they prioritized nine lncRNAs termed lncRNA Glioma Radiation Sensitizers (lncGRS), with lncGRS-1 being the top hit [36]. Knockdown of *lncGRS-1* (*CTC-338 M12.4*) inhibited the growth of glioma cell lines and tumor growth in human brain organoids but did not affect normal brain cells. Mechanistically, lncGRS-1 knockdown activated the p53 signaling pathway and cell cycle arrest genes like *CDKN1A* and sensitized glioma cells to radiation-induced DNA damage markers γH2AX and p53BP1.

### 5.3. Responsiveness to Chemotherapy

Chemotherapy is a common treatment for GBM, but many GBM tumors develop resistance to chemotherapy, leading to treatment failure and disease progression. CRISPR screening has been employed to elucidate the genetic basis of drug sensitivity and uncover potential vulnerabilities that can be targeted to overcome resistance.

Temozolomide (TMZ) is the primary chemotherapy drug used for GBM treatment [5,15]. MacLeod et al. conducted genome-wide CRISPRn screens in a series of patient-derived GSCs and identified multiple modulators of TMZ sensitivity [44]. They showed that knockout of genes in the mismatch repair (MMR) pathway, including *MLH1*, *MSH2*, *MSH6*, and *PMS2*, leads to TMZ resistance in GSCs, whereas knockout of genes in the Fanconi anemia/interstrand crosslink repair pathway (such as *FANCA* and *C19orf40*) and homologous recombination pathway (such as *MCM8* and *MCM9*) sensitizes GSCs to TMZ. In another study, Rocha et al. performed genome-wide CRISPRn and CRISPRa screens in GBM cell lines under TMZ selection and validated roles for DNA repair genes like *MSH2* in conferring resistance^55^, as has been identified in the previous study [44]. They also identified new resistance pathways involving Sonic Hedgehog, circadian rhythm genes, the NRF2 antioxidant response, and Wnt/β-catenin signaling [57]. Genes in these pathways, including *CLCA2*, *PTCH2*, *FZD6*, and *CTNNB1*, were shown to promote cell survival when overexpressed, suggesting they could be targeted to improve TMZ efficacy. They also showed that *NRF2* can be regulated by clock genes and promote TMZ resistance by regulating glutamate–cysteine ligases.

Several additional studies have focused on TMZ resistance in GBM cells with specific genetic backgrounds. The study by Huang et al. focused on TMZ sensitivity in GBM carrying the EGFRvIII mutation. Through a genome-wide CRISPRn screen, they uncovered *E2F6* as a key factor promoting TMZ resistance in EGFRvIII GBM cells. The expression of *E2F6* is regulated by the EGFRvIII/AKT/NF-κB pathway and can be used as a predictive marker for TMZ response in patients [58]. The screen also identified *MUC1* as an essential gene for TMZ resistance in EGFRvIII GBM, and a follow-up study revealed that MUC1, specifically its cleaved C-terminal subunit MUC1-C, mediates TMZ resistance by stabilizing EGFRvIII through evasion of lysosomal degradation [59]. A recent study by Cheng et al. focused on genetic modulators of TMZ sensitivity in *RAD18*^−/−^ GBM cells. They discovered that RAD18, an E3 ubiquitin ligase, is activated in TMZ-treated GBM cells and promotes TMZ resistance. Through CRISPRn screens targeting DNA damage response (DDR)-related genes, they found that knockout of MMR genes, including *MLH1*, *MSH6*, *PMS1*, *PMS2*, and *MSH2*, leads to TMZ resistance in both WT and *RAD18*^−/−^ GBM cells, consistent with other studies [44,57], whereas knockout of genes in other DDR pathways, including *POLD3*, *CHEK2*, and *PRKDC*, preferentially sensitizes *RAD18*^−/−^ GBM cells to TMZ treatment [60].

In addition to TMZ, CRISPR screens have been applied to profile the responsiveness of GBM to other chemotherapy drugs. Etoposide is a topoisomerase II inhibitor that is used in chemotherapy to treat a variety of cancers by inducing DNA damage [61]. To identify genes that influence GBM responsiveness to etoposide, Awah et al. performed a genome-scale CRISPRn screen in GBM cells treated with etoposide [62]. By overlapping the screen hits with genes whose expression correlates with drug response in other cell lines, they identified the ribosomal proteins RPS11, RPS16, and RPS18. Further experiments showed that knockout of *RPS11* led to resistance by impairing the induction of the pro-apoptotic gene *APAF1* in response to etoposide and doxorubicin treatments.

RSL3 is a small molecule that induces ferroptosis, a recently characterized form of regulated cell death showing potential as a new therapeutic strategy against tumors. The genetic determinants of RLS vulnerability in GBM remained unclear. Cao et al. performed a genome-wide CRISPRn screen in GBM cells treated with RSL3. Combining RNA sequencing of RSL3-resistant cells, they identified ALOX15, a lipoxygenase enzyme, as an essential driver of ferroptosis [63]. Small activating RNA (saRNA) was used to upregulate *ALOX15* and induce ferroptosis [63]. Macrophage membrane-coated nanoparticles loaded with saALOX15 (Ang-MMsaNPs) were developed for targeted GBM therapy. Ang-MMsaNPs induce ferroptosis by promoting mitochondrial damage and dysfunction, as shown by transcriptomic and functional analyses.

CRISPR screening has also been employed to identify targets of chemotherapy drugs. For example, Qu et al. developed gambogic amide (GA-amide), a potential new chemotherapy drug for GBM that can effectively penetrate the blood–brain barrier and inhibit tumor growth [64]. They conducted a genome-wide CRISPRn screen to identify the target of GA-amide. Their results revealed *WD repeat domain 1 (WDR1)* as the direct binding target of GA-amide. Follow-up work showed GA-amide suppresses GBM by disrupting cytoskeletal homeostasis and activating the mitochondrial apoptosis pathway via inhibition of *WDR1*.

### 5.4. Responsiveness to Immunotherapy

Immunotherapy has emerged as a promising new approach for tumor treatment, including GBM [14,18]. As GBM tends to be relatively immune-privileged, evading immune detection and clearance, immunotherapies aim to activate or enhance the immune response against the tumor [9]. Several strategies are under investigation, including checkpoint inhibitors that block immune-suppressive pathways like PD-1/PD-L1 [65], chimeric antigen receptor T (CAR T)-cell therapies targeting tumor-specific antigens [23,66], and cancer vaccines [4] to stimulate an anti-tumor immune response.

Natural killer (NK) cells and T cells are two types of important immune cells in the tumor microenvironment that can directly recognize and kill tumor cells [67,68]. NK cells are a part of the innate immune system and can induce tumor cell death through cytotoxic granules or death receptors without prior sensitization [68,69]. CD8^+^ T cells are cytosolic lymphocytes of the adaptive immune system that kill tumor cells through T-cell receptor recognition of cancer antigen peptides presented on MHC class I molecules [67,70].

Two complementary studies employed CRISPR screening to identify mechanisms regulating GBM evasion of NK cells and T cell-mediated killing, respectively. In the first study, Bernareggi et al. performed a genome-wide CRISPRn screen in GSCs challenged with NK cells [69]. They identified CHMP2A, a component of the ESCRT-III complexes, as a top hit that increased GSC sensitivity to NK cells when knocked out. A key mechanism found was that *CHMP2A* deletion activated NF-κB signaling and increased secretion of chemokines like CXCL10 and CXCL12, promoting NK cell migration towards tumor cells [69]. *CHMP2A* was also found to mediate tumor resistance by secreting extracellular vesicles containing ligands like MICA/B and tumor necrosis factor-related apoptosis-inducing ligand (TRAIL) that induce NK cell apoptosis [69].

The second study by Dmello et al. focused on the GBM response to CD8^+^ T cells [71]. They conducted an in vivo CRISPRn screen targeting 713 kinases in a xenograft glioma model in WT and CD8^+^ KO mice. They identified checkpoint kinase 2 (Chek2) as the most important kinase mediating GBM resistance to CD8 T cell killing. Its knockout was found to increase PD-L1 expression in mouse glioma cells in response to IFNγ through activation of the STING pathway. Combining pharmacological inhibition of Chek1/2 with PD-1 or PD-L1 blockade improved survival in mouse glioma models.

CAR T-cell therapy, which utilizes genetically engineered patient-derived T cells to target and eliminate tumor cells, has emerged as a transformative approach to cancer treatment [66]. However, its efficacy in solid tumors, including GBM, needs further optimization [66,72]. To identify novel targets that could enhance the cytotoxicity of CAR T cells against GBM, Wang et al. performed whole-genome CRISPRn screens on both CAR T cells and GSCs in a co-culture system [23]. They found that knocking out targets in GSCs, such as *RELA* and *NPLOC4*, sensitized them to CAR T cell-mediated killing [23]. In addition, knocking out certain targets in CAR T cells, such as *TLE4* and *IKZF2*, potentiated their long-term activation, cytolytic activity, and in vivo antitumor function against GSCs [23].

Similarly, Larson et al. conducted a genome-wide CRISPRn screen in U87 cells exposed to EGFR-targeting CAR T cells and identified that loss of genes in the interferon-gamma (IFNγ) receptor signaling pathway, including *IFNGR1*, *JAK1*, and *JAK2*, rendered GBM cells more resistant to CAR T cell cytotoxicity both in vitro and in vivo [66]. Mechanistically, IFNGR1 depletion reduced ICAM-1 expression and EGFR-CAR T cell cytotoxicity. They demonstrated that IFNγ receptor signaling was required for sufficient adhesion of CAR T cells to GBM cells.

Finally, Li et al. conducted a CRISPRi screen targeting kinases and drug target genes in GBM cells co-cultured with B7-H3 CAR T cells [73]. They discovered that knocking down *ARPC4* or *NDUFV1* in GBM increases CAR T cell-mediated killing through upregulating the immunostimulatory factor TNFSF15, which promotes CAR T cell activation and cytotoxic effector molecule production.

## 6. Discussion and Future Directions

CRISPR-based genetic screening has significantly advanced our understanding of GBM vulnerability, leading to the identification of potential new targets and corresponding drugs for GBM treatment, such as DOT1L inhibitor EPZ-5676 [44] and Check1/2 inhibitor Prexasertib [71,74] (Table 1).

However, several challenges remain to be addressed in future development. Most screens to date have relied on established GBM cell lines, which do not fully capture tumor heterogeneity. Patient-derived GSC models [24,43,44,45] are an improvement but still lack microenvironment complexity [67]. Advanced in vivo screening strategies that directly interrogate tumor genetics in clinically relevant models are needed. Recently developed approaches like AAV-mediated direct brain delivery [42] or tumor organoid transplantation [71,76] hold promise for more physiological screening but require further scale-up and optimization. Novel platforms that enable CRISPR screens directly in patient-derived samples, such as PDX models, could enable personalized target discovery and validation.

While many genetic dependencies and pathways have been elucidated, most existing studies have profiled only a limited number of patient samples. Key vulnerabilities identified are occasionally specific to a single sample, limiting broader clinical translation [14]. Larger consortium-based screening efforts of diverse tumor subtypes are needed to define core conserved vulnerabilities across the heterogeneous landscape of GBM.

CRISPR technology is rapidly advancing, and new methods are emerging that allow for more sophisticated screening in GBM. For example, the development of multiplex CRISPR screening methods will allow for the simultaneous screening of multiple genes or pathways [77], which may uncover more complex biological networks driving GBM progression and resistance. While most screens to date relied on gene-level perturbations introduced by CRISPRn, CRISPRi, or CRISPRa, other CRISPR-based tools, such as base editor and prime editor, can be used to assess the effect of specific disease-associated mutations in GBM [28,32].

CRISPR screening can also be integrated with other technologies, such as single-cell sequencing, to provide a more comprehensive understanding of the molecular mechanisms underlying GBM. For example, single-cell CRISPR screening can be used to reveal transcriptome changes in response to genetic perturbations in specific subpopulations within GBM tumors [23,78].

## 7. Conclusions

CRISPR/Cas9-based screens have provided invaluable insights into the molecular mechanisms underlying GBM tumorigenesis, growth, invasion, and resistance to radiotherapy, chemotherapy, and immunotherapy. These studies have identified novel targets and pathways that could be exploited for the development of more effective therapies for GBM. As CRISPR technology and GBM models continue to advance and new applications are developed, it is anticipated that CRISPR screening will play an increasingly important role in the discovery of novel targets and the development of more effective therapies for GBM.

## Figures and Tables

**Table 1:** Current studies using CRISPR-based screens in GBM models.

Phenotype	Cell Model	CRISPR System	Screening Condition	sgRNA Library	Main Findings	Ref.
GBM tumorigenesis	astrocytes	CRISPRn	AAV delivery of sgRNA library into the brain of LSL-Cas9 transgenic mice	mTSG library: 286 sgRNAs targeting 56 tumor suppressor genes	determined mutational profiles in GBM tumorigenesis; identified co-occurring driver combinations like *B2m-Nf1* and *Zc3h13-Rb1*	[42]
GBM growth	U87	CRISPRn	normal condition, without treatment	Custom library: 557 sgRNA targeting 557 E3 ligase genes	identified RNF185 as a tumor suppressor regulated by miR-587	[75]
	U87	CRISPRi	implanted in the brain of NU/J mice	CRinCL—Unique to U87: 23,317 sgRNAs targeting 2307 lncRNAs	identified 17 lncRNA essential for GBM growth in vivo	[48]
	U87, U251	CRISPRi	normal condition, without treatment	Custom library: 9083 sgRNAs targeting 1209 lncRNAs dysregulated in GBM cells	*DARS1-AS1* promotes GBM growth through interaction with YBX1	[49]
	T98G-TERT-ON GBM cells	CRISPRn	normal condition, without treatment	Custom Library: sgRNAs targeting *AAVS1*, *TERT* and *GABPB1L*	did not detect genetic vulnerabilities specific to GBM carrying TERT promoter mutations (TPMs)	[54]
	T98G, U373	CRISPRn	normal condition, without treatment	EpiDoKOL: 1628 sgRNA targeting 251 chromatin modifiers	*ASH2L* is essential for GBM cell survival by regulating cell cycle, transcription, and histone methylation through interactions with histone methyltransferases	[46]
	patient-derived GSCs, NSCs	CRISPRn	normal condition, without treatment	GeCKO library: 64,751 sgRNAs targeting 18,080 genes	knockout of *PKMYT1* specifically inhibits the growth of GSCs by impairing cell division	[43]
	patient-derived GSCs	CRISPRn	normal condition, without treatment	Custom library: targeting 160 chromatin regulator genes from ChIP-seq profiling	knockout of *YY1* inhibits the proliferation and self-renewal of GSCs by controlling transcription and m6A modification	[24]
	patient-derived GSCs, NSCs	CRISPRn	normal condition, without treatment	TKOv1/TKOv3 library: 70,948 sgRNAs targeting 18,053 genes	identified transcription factors *SOX2* and *SOX9*, histone methyltransferase *DOT1L*, and cytokine signaling suppressor *SOCS3* as important regulators of GSC stemness and fitness	[44]
	patient-derived GSCs	CRISPRn	cultured as spheres or in the 3D bioprinted tissue model	Brunello library: 76,441 sgRNAs targeting 19,114 genes	identified *PAG1*, *ZNF830*, *ATP5H*, *RNF19A* as essential genes of GSCs	[45]
GBM invasion	U138	CRISPRn	cultured in a transwell system	Custom library: 45,740 sgRNAs targeting 4574 genes relative to cell motility and drug targets	knockout of *MAP4K4* reduces invasion and inhibits mesenchymal transition of GBM cells	[51]
	patient-derived GSCs	CRISPRn	cultured in 3D hydrogel invasion devices	Custom library: 29,790 sgRNAs targeting 2981 metabolic genes	knockdown or inhibition of *CTH* impaired GBM invasion in vitro and in vivo and caused cysteine deficiency and ROS accumulation	[52]
GBM responsiveness to radiotherapy	U87	CRISPRi	treated with radiation	CRiNC-U87 and HEK293T and CRiNCL-Unique to U87:38,011 sgRNAs targeting 3750 lncRNAs	knockdown of *lncGRS-1(CTC-338 M12.4)* selectively inhibits GBM growth and enhances GBM sensitivity to radiation	[36]
	U87, U251	CRISPRa	treated with radiation	SAM library: 70,290 sgRNAs targeting 23,430 genes	CARHSP1 enhances radiation resistance in GBM via TNF-α/NF-kβ pathway	[56]
GBM responsiveness to chemotherapy	U138	CRISPRn	treated with TMZ	GeCKO v2 library: 123,411 sgRNAs targeting 19,050 genes	knockout of *MSH2*, *MSH6*, *CLCA2* or *PTCH2* enhances TMZ resistance	[57]
	U138	CRISPRa	treated with TMZ	SAM library: 70,290 sgRNAs targeting 23,430 genes	NRF2 enhances TMZ resistance by controlling the expression of enzymes in GSH synthesis	[57]
	*RAD18^+/+^* and *RAD18^−/−^* U373 cells	CIRSPRn	treated with TMZ	DDR-CRISPR lentivirus library: 5040 sgRNAs targeting 504 DDR genes	knockout of *POLD3* leads to greater TMZ sensitivity in *RAD18*-deficient GBM cells	[60]
	WT and EGFRvIII U87 cells	CRISPRn	treated with TMZ	GeCKO v2: library123,411 sgRNAs targeting 19,050 genes	E2F6 enhances TMZ resistance by promoting DNA repair	[58]
	WT and EGFRvIII U87 cells	CRISPRn	treated with TMZ	GeCKO v2 library: 123,411 sgRNAs targeting 19,050 genes	MUC1 enhances TMZ resistance by regulating DSB repair and autophagy	[59]
	patient-derived GSCs	CRISPRn	treated with TMZ	TKOv1/TKOv3 library: 70,948 sgRNAs targeting 18,053 genes	Knockout of genes in the MMR pathway, including *MLH1*, *MSH2*, *MSH6*, and *PMS2*, leads to TMZ resistance in GSCs; knockout of genes in the FA and HR pathways (such as *FANCA*, *MCM8*, and *MCM9*) sensitizes GSCs to TMZ	[44]
	LN229	CRISPRn	treated with RSL3	GeCKO v2 library: 123,411 sgRNAs targeting 19,050 genes	identified *ALOX15* as an essential driver of ferroptosis in GBM	[63]
	SNB19, U251, patient-derived GBM cells	CRISPRn	treated with etoposide	Brunello Library: 76,441 sgRNAs targeting 19,114 genes	knockout of *RPS11* reduces GBM responsiveness to etoposide by impairing the induction of the pro-apoptotic gene *APAF1*	[62]
	patient-derived GSCs	CRISPRn	treated with Gambogic amide (GA-amide)	Brunello Library: 76,441 sgRNAs targeting 19,114 genes	*WDR1* is the direct binding target of GA-amide, a potential new chemotherapy drug for GBM	[64]
GBM responsiveness to T cell cytotoxicity	U87	CRISPRn	co-cultured with EGFR-targeting CAR T cells	Brunello library: 76,441 sgRNAs targeting 19,114 genes	knockout of genes in the IFNγ signaling pathway, including *IFNGR1*, *JAK1* and *JAK2*, induces GBM resistance to CAR T cell cytotoxicity	[66]
GBM responsiveness to T cell cytotoxicity	patient-derived GSCs	CRISPRn	co-cultured with IL13Rα2-targeting CAR T cells	Brunello library: 76,441 sgRNAs targeting 19,114 genes	knockout of *RELA* or *NPLOC4* sensitizes GBM to CAR T cell-mediated killing	[23]
	U87, U251 and T98G	CRISPRi	co-cultured with B7-H3 targeting CAR T cells	H1 library: 13,025 sgRNAs targeting 2318 genes of kinases, phosphatases, and drug target	knockdown of *ARPC4* or *NDUFV1* in GBM cells enhances their killing by CAR T cells by activating TNFSF15-mediated cytokine signaling pathways.	[73]
	GL261	CRISPRn	implanted in WT and CD8 KO mice	Brie kinome KO library: 2856 sgRNA targeting 714 kinases	identified Chek2 as the most important kinase mediating GBM resistance to CD8^+^ T cell killing	[71]
GBM responsiveness to NK cell cytotoxicity	patient-derived GSCs	CRISPRn	co-cultured with NK cells	Brunello library: 76,441 sgRNAs targeting 19,114 genes	knockout of *CHMP2A* in GSCs sensitizes them to NK cells by activating NF-κB signaling and increasing the secretion of chemokines like CXCL10 and CXCL12	[69]

mTSG library: mouse homolog tumor suppressor gene library; GeCKO library: genome-scale CRISPR-Cas9 knockout library; Brunello library: the Human CRISPR Knockout Pooled Library; EpiDoKOL: Epigenetic Domain-specific Knockout Library; SAM library: Human CRISPR activation library; CRiNCL: CRISPRi Non-Coding Library; WT, wildtype; MMR, mismatch repair; FA, Fanconi anemia; HR, homologous recombination; DDR, DNA damage response.

## Abbreviations

AAV	adeno-associated virus
CAR T	chimeric antigen receptor T
Chek2	checkpoint kinase 2
CRiNCL	CRISPRi Non-Coding Library
CRISPR	Regularly Interspaced Short Palindromic Repeats
CRISPRa	CRISPR activation
CRISPRi	CRISPR interference
CTH	cystathionine gamma-lyase
dCas9	inactive Cas9, dead Cas9
DDR	DNA damage response
DSBs	double-strand breaks
EpiDoKOL	Epigenetic Domain-specific Knockout Library
FACS	fluorescence-activated cell sorting
GA-amide	gambogic amide
GBM	Glioblastoma, Glioblastoma multiforme
GeCKO	genome-scale CRISPR-Cas9 knockout
GSCs	Glioblastoma stem cells, GBM stem-like cells
HR	homologous recombination
KD	knockdown
KO	knockout
lncGRS	lncRNA Glioma Radiation Sensitizers
lncRNAs	long non-coding RNAs
MGMT	methyl guanine methyl transferase
mTSG	mouse homolog tumor suppressor gene
nCas9	Cas9 nickase
NGS	Next-generation sequencing
NHEJ	non-homologous end joining
NSCs	neural stem cells
PDX	patient-derived xenograft
pegRNA	prime editing guide RNA
RNAi	RNA interference
saRNASmall	activating RNA
sgRNA	single guide RNA
shRNA	short hairpin RNA
siRNA	small interfering RNA
TERT	telomerase reverse transcriptase
TMZ	temozolomide
TPMs	telomerase reverse transcriptase promoter mutations
TRAIL	tumor necrosis factor-related apoptosis-inducing ligand
WDR1	WD repeat domain 1
